# Applicability of anthropometric indicators to assess physical fitness: proposal of percentiles for schoolchildren living at high altitude in Peru

**DOI:** 10.1016/j.jped.2025.101497

**Published:** 2026-01-12

**Authors:** Jose Fuentes-Lopez, Ruben Vidal-Espinoza, Danilo Rodrigues Pereira da Silva, Dony Mamani-Velásquez, Eliseny Vargas-Ramos, Estanislao Pacompia-Cari, Wilbert Cossio-Bolaños, Marco Cossio-Bolaños, Rossana Gomez-Campos

**Affiliations:** a Universidad Nacional del Altiplano, Puno, Perú; b Universidad Católica Silva Henriquez, Santiago, Chile; c Universidade Federal de Sergipe, Departamento de Educação Física, São Cristóvão, SE, Brazil; d Universidad Privada San Juan Bautista, Escuela Profesional de Estomatologia, Segunda Especialidad, Lima, Perú; e Universidad Católica del Maule, Talca, Chile

**Keywords:** Anthropometric indicators, Physical fitness, Children, Percentiles, Altitude

## Abstract

**Objective:**

a) To verify the applicability of anthropometric indicators to evaluate physical fitness in school children and b) Propose physical fitness reference values for Peruvian children living at high altitudes in Peru.

**Methods:**

A cross-sectional study was carried out in children aged 6 to 12 years living at high altitude in Peru. A total of 1372 primary school children (785 males and 587 females) were evaluated. Weight, height and four physical tests [Hand grip strength (right and left HGS)], Horizontal jump (HJ), Round trip (5 × 10 repetitions), and the 6-minute walk test (6MWT)] were evaluated. Body Surface Area (BSA), Body Mass Index (BMI), and Triponderal Index (TPI) were calculated.

**Results:**

The BSA presented a better comparative structure in relation to BMI and TPI with all physical tests. In males, HGS was related between *r* = 0.64 and 0.66; HJ (*r* = 0.26), agility (*r* = -0.31) and 6MWT (*r* = 0.30). In females, HGS was related between *r* = 0.63 and 0.64; HJ (*r* = 0.36), agility (*r* = -0.36), and 6MWT (*r* = 0.21). The predictive power in the four physical tests ranged from R^2^ = 9% to 43% in males, and from 5% to 41% in females. Percentiles by age and sex were proposed for BSA, HGS (right and left), HJ, agility, and 6MWT.

**Conclusion:**

BSA is the best predictor of physical fitness in schoolchildren from high altitude areas in Peru, surpassing BMI and TPI. In addition, reference values were proposed to evaluate physical fitness in these children.

## Introduction

Physical fitness assessment has become an elementary indicator of the health status of children and adolescents.^1^ It is often assessed by several tests or test batteries that measure different aspects of fitness or components.^2^ They are generally represented by five components: morphological, muscular, motor, cardiorespiratory and metabolic.^3^

The assessment and monitoring of physical fitness at the school level is relevant. It serves not only to establish physical performance categories, but also to set health goals, plan lasting healthy behaviors, and boost physical education teaching among schoolchildren.^4^

Indeed, in the school system, it is important for schools to implement batteries of health-related fitness tests. Especially that they are age-appropriate and reflect as well as possible the relationship between physical fitness and health.^5^

In this sense, it is widely known that the use of anthropometric indicators (morphological components) is relevant in the school population. Since it allows the monitoring of somatic growth and development^6^ and serves as a useful tool for risk identification, intervention, or impact assessment on nutritional status or health in general.^7^

These anthropometric indicators [e.g. body mass index (BMI), triponderal index (TPI), waist height index (WHI), waist circumference (WC), among others), have been used by several studies as predictors of various physical tests in children and adolescents.^8^,^9^ However, the relationships of these variables were weak, suggesting that they might not be the most appropriate and accurate predictors of physical performance in schoolchildren.

These indicators often do not fully capture variability in body size and proportion, factors that can influence capacity and efficiency in physical performance. In addition, other previous studies have shown that children and adolescents living in regions of moderate and/or high altitude have evidenced shorter stature compared to international standards.^10^,^11^ This fact suggests a possible effect of the hypoxic environment on linear growth, since it has been postulated that altitude influences physical growth parameters in residents of high-altitude areas due to continuous exposure to hypoxia.^12^ As a consequence, when relating weight to height, these children tend to have higher BMI and TPI values.

This could lead to an overestimation of excess weight when using this indicator in high-altitude populations. Therefore, this phenomenon, together with the weak relationships observed between anthropometric indicators and physical performance tests in previous studies, highlights the need to consider other anthropometric parameters, such as body surface area (BSA). This indicator could correct or improve such relationships. Since it provides a more accurate estimate of total body size, which varies significantly during growth and development throughout age (e.g., increases from 0.2 m² at birth to approximately 1.73 m² in adulthood).^13^

Therefore, the objectives of the study are: a) to verify the applicability of anthropometric indicators for assessing physical fitness in schoolchildren, and b) to propose physical fitness reference values for Peruvian children living at high altitude in Peru.

## Materials and methods

### Type of study and sample

A descriptive cross-sectional study was carried out in 1372 primary school children (785 males and 587 females). The age range was 6 to 12 years. The sample selection was non-probabilistic (convenience). Data were collected in 4 primary schools in the urban area of the city of Puno. This city is located at 3820 m above sea level and is geographically located in the southeast of the country (Peru) and is bordered to the east by the department of La Paz (Bolivia).

**Figure 1:**
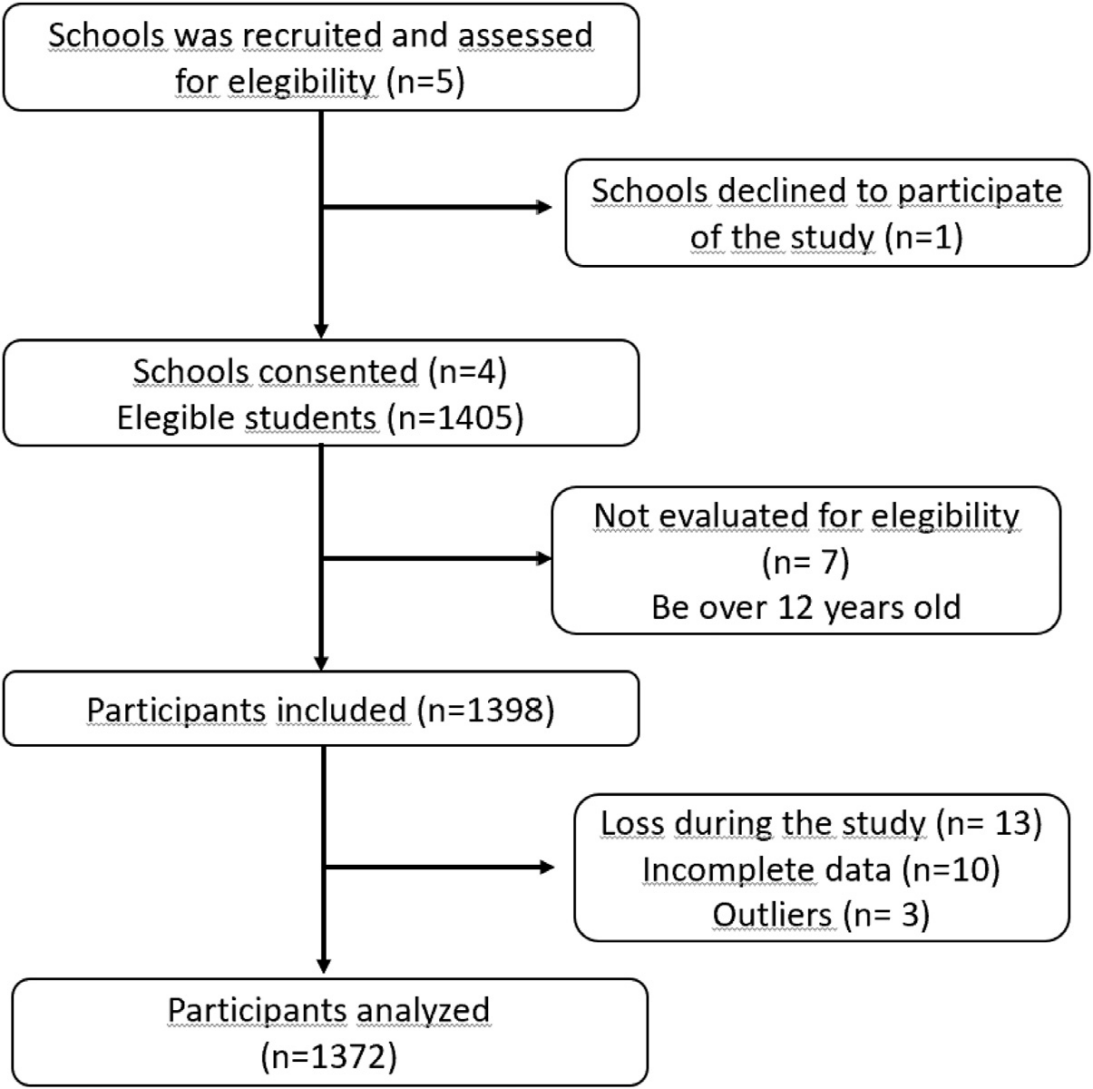
Flowchart of the study sample selection process.

The study included children of both sexes who attended physical education classes and were enrolled at the primary level and aged 6 to 12 years. Children who did not complete the evaluation of anthropometric measurements and physical tests were excluded. As well as those with physical and/or sports injuries (Figure 1).

The study was performed according to the approval of the ethics committee of the National University of the Altiplano of Puno (CEIC 007–2022) and according to the ethical principles of the Declaration of Helsinki for human beings.

### Techniques and procedures

The four schools were invited to participate voluntarily in the study, and once participation was accepted, informed consents were sent. Data collection took place from April to June 2023.

A team of six evaluators with ample experience in anthropometric and physical evaluations was formed and went to the schools to collect the data. In anthropometry, weight and height were evaluated, and in physical fitness, four physical tests were evaluated: Hand grip strength (HGS), right and left, Horizontal jump (HJ), Agility (5 × 10 repetitions), and the 6-minute walk test (6MWT).

### Anthropometric variables

Weight and height were evaluated with the least amount of clothing possible (barefoot, shorts, and T-shirt) according to the suggestions described by Ross and Marfell-Jones.^14^ For weight (kg), a Tanita digital scale (United Kingdom, Ltd.) with an accuracy of 0.1 kg and a range of 0.1 kg to 150 kg was used. For height, a portable stadiometer (Hamburg, Seca, Ltd.) with an accuracy of 0.1 mm and a measurement range of 0.0 cm to 220.0 cm was used. With both measurements (Weight and Height) BMI: [BMI = weight (kg)/height (m)^2^], TPI: [TPI = weight (kg)/height (m)^3^], and BSA: BSA = 0.024265 × weight (kg)0.5378 × height (cm)0.3964 were calculated. The BSA was determined by the equation proposed by Haycock et al.^15^

### Physical fitness

**Hand grip strength (HGS):** The HGS of both hands (right and left) was evaluated. It was evaluated according to the suggestions described by Richards et al.^16^ The evaluee sits on a straight-backed chair. Then one of the evaluators constantly adjusted the dynamometer to the grip size of the equipment according to age and sex. A JAMAR hydraulic dynamometer (Hydraulic Hand Dynamometer ® Model PC-5030 J1, Fred Sammons, Inc., Burr Ridge, IL: USA) was used for the measurement. This equipment has an accuracy of 0.1 kg and a scale of up to 100 kg/f.

**Horizontal jump HJ (m):** The evaluated is located behind a previously drawn line. It was evaluated according to the suggestions described by Castro-Piñero et al.^17^ The subject must perform preparatory movements of arms and knees to execute the jump. Once the jump is performed, the distance at the end of the heel is recorded. A metallic tape measure was used to record the distance from 0 to 3 m.

**Round trip run (5 m** **× 10 repetitions) (seconds):** It was evaluated using the suggestions described by Verschuren et al.^18^ The person evaluated should go in a back-and-forth direction, covering a distance of 5 m until completing a total of 50 m At the end, 10 repetitions were performed. A Casio digital stopwatch with an accuracy of 0.01 s was used to measure the time.

**6-minute walk (6MWT):** This was performed according to the recommendations of the American Thoracic Society.^19^ It was performed in an open space on a flat surface (30 m long × 10 m wide). This area was demarcated with white adhesive tape to distinguish the lanes. The evaluators instructed the schoolchildren to walk as fast as they could in one direction, back and forth as fast as they could (without stopping). At the end of the 6 min, the distance achieved by each child was recorded.

The reliability of the physical tests was controlled through the test-retest technique. It was applied to 10 % of the sample (*n* = 142). The values of the relative technical error of measurement (TEM %) ranged from 1.0 to 1.6 %.

### Statistics

The Kolmogorov-Smirnov test was used for the normal distribution of data. Next, the data analyzed descriptively were: (mean, standard deviation, and range). To examine the relationships between anthropometric indicators and physical fitness, Pearson's correlation coefficients (r) were calculated to evaluate the linear associations between BMI, TPI, BSA, and the four physical tests.

In addition, a linear regression analysis was performed to determine the predictive power of anthropometric indicators on physical fitness. The coefficient of determination (r²) was calculated to quantify the proportion of variance in fitness test performance explained by the anthropometric indicators. The significance considered in all cases was *p* < 0.05. Statistical analyses were performed in Excel spreadsheets and SPSS 18.0.

## Results

Table 1 shows the anthropometric variables and physical tests characterizing the sample studied. In body weight, there were no differences between both sexes (*p* > 0.05) from 6 to 12 years of age. In height, males showed greater height at 6 and 12 years of age in relation to females (*p* < 0.05). In the other ages, there were no significant differences. In the anthropometric indicators (BMI, TPI and BSA), there were no significant differences between both sexes and at all ages (6 to 12 years) (*p* > 0.05). In the HGS of both hands and the 6MWT test, there were only significant differences at 12 years of age between both sexes (*p* < 0.05). In the other ages, from 6 to 11 years of age, there were no significant differences. In the HJ, males presented better results at all ages (*p* < 0.05), except at 11 years of age, where there were no significant differences (*p* > 0.05). In the agility test, males showed better results in relation to their female counterparts at all ages (*p* < 0.05).

**Table 1:** Anthropometric and physical characteristics of the schoolchildren studied.

Age (years)	n	Weight (kg)		Height (cm)		BMI (kg/m^2^)		TPI (kg/m3)		BSA (cm2)		HGSR (kgf)		HGSL (kgf)		Horizontal jump (cm)		Agility 5 × 10 (seg)		6MWT	
		X	SD	X	SD	X	SD	X	SD	X	SD	X	SD	X	SD	X	SD	X	SD	X	SD
Males																					
6	59	24.5	6.4	119.6*	6.6	17.1	4	14.4	3.3	0.88	0.17	6.5	1.7	6.4	1.7	83.2*	21.8	29.5*	3.4	493.5	48.2
7	110	26.2	5	122.9	6.4	17.2	2.5	14.1	2	0.94	0.11	7.3	2.1	7.1	1.9	91.1*	21.7	28.0*	3.7	508.7	55
8	123	30.7	6.5	128.5	5	18.5	3.1	14.4	2.3	1.04	0.13	8.8	2.6	8.6	2.7	99.3*	16.1	26.2*	3	540.1	57.8
9	112	34.1	8.6	133.2	6.3	19.1	3.8	14.3	2.7	1.12	0.16	10.8	3.7	10.3	3.5	103.7*	23.7	24.7*	2.3	551.1	65.8
10	131	37.5	9	138.1	6	19.4	3.9	14.1	2.5	1.2	0.17	13.1	4	13.2	4.3	116.8*	20.3	23.7*	2.3	583.8	56.8
11	135	41.6	9.6	143.9	7	20	3.8	13.9	2.5	1.29	0.17	15.1	3.8	14.3	3.6	118.5	22.3	23.3*	2.3	605.3	70
12	115	46.6	10.7	151.0*	6.84	20.4	3.96	13.6	2.55	1.39	0.18	19.0*	5.5	18.4*	5.23	132.0*	24.6	22.1*	1.86	624.5*	68.3
Females																					
6	56	22.7	8	116.2	3.9	16.8	5.9	14.5	5.1	0.85	0.14	6	1.8	5.5	1.4	77.2	15.3	29.5	3.5	501.6	54.2
7	91	25.4	7.5	121.6	5.3	17.2	4.7	14.1	3.8	0.92	0.13	7.2	1.8	6.7	2	82.8	17.1	29.5	3.5	535.4	52
8	112	28.1	5.4	127.3	5.3	17.3	2.6	13.6	1.9	0.99	0.11	8.7	4.3	8.2	4.1	94	18.1	27	2.5	557.0	53.4
9	98	32.4	8.2	132.6	6.5	18.3	3.7	13.8	2.7	1.09	0.15	10.3	2.4	9.5	2.9	96.9	16.5	25.9	2.3	560.3	59.9
10	98	37.1	6.7	140.6	6	18.7	2.9	13.3	2.2	1.2	0.13	13.3	4.1	12.6	3.7	104.8	17	24.8	1.8	588.4	78.1
11	77	41.5	9.7	145.3	6.5	19.5	3.5	13.4	2.2	1.29	0.17	15.8	3.8	15.2	4.3	117.4	17	24.4	2.3	604.5	60.6
12	55	45.7	9	148.3	6.4	20.6	3.1	13.9	1.9	1.37	0.16	17.9	3.8	16.5	3.9	115	16.2	23.9	1.7	581	60.6

HGSR, Right hand grip strength; HGSL, Left hand grip strength; HJ, Horizontal jump; 6MWT, 6-minute walk test; SEE, Standard error of estimation; BMI, Body mass index; TPI, Triponderal index; BSA, Body surface area; X, Mean; SD, Standard deviation.

**Figure 2:**
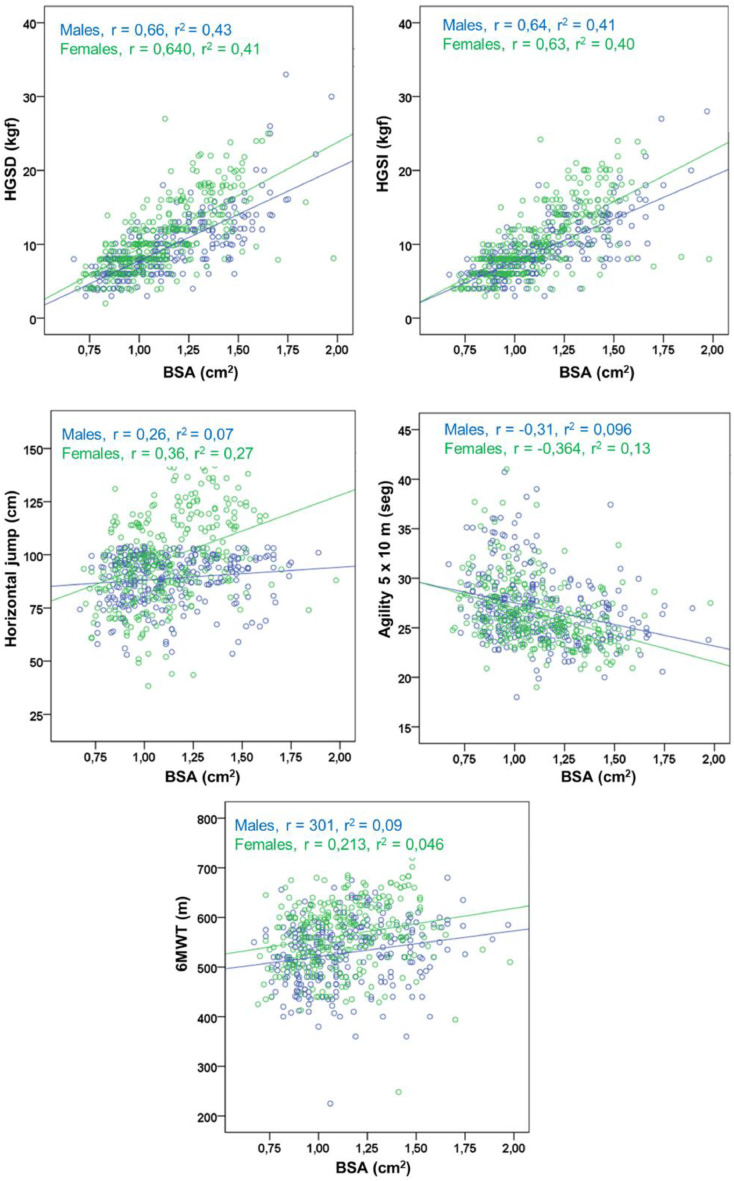
Relationship between BSA and physical fitness tests in schoolchildren aged 6 to 12 years of both sexes.

Table 2 shows the correlation coefficients, determination, SEE, and regression parameters (intercept and slope) between anthropometric indicators (BMI, TPI and BSA) with four physical tests in both sexes. BMI correlated with HGS (right and left), showing values of *r* = 0.35 in males and *r* = 0.27 in females. Meanwhile, with the HJ, Round trip run (5 m × 10 repetitions) and 6MWT tests, the relationships in both sexes were null (*r* < 0.06). In the case of TPI, paradoxically, there was no relationship with HGS (right and left) in both sexes (*r* < 0.01). However, the relationships improved with the rest of the physical tests in both sexes. In males, the HJ was *r* = 0.29, agility *r* = −0.26 and 6MWT *r* = 0.24 and in females, the HJ was *r* = 0.14, agility *r* = −0.15, and 6MWT *r* = 0.20. The relationships between the BSA and the four physical tests in both sexes improved ostensibly. In males, HGS with both hands ranged from *r* = 0.64 to 0.66, with HJ *r* = 0.26, agility *r* = −0.31, and with 6MWT *r* = 0.30. In females, HGS with both hands is related from *r* = 0.64 to 0.63, with HJ *r* = 0.36, agility *r* = −0.36, and with 6MWT *r* = 0.21. Figure 2 shows the explanatory power (R^2^) between BSA and physical tests in both sexes. In males, the values ranged from 9 to 43 %, and in females, from 5 to 41 %.

**Table 2:** Correlations and regression between anthropometric indicators (BMI, TPI, BSA) with physical fitness tests by sex.

Variables	BMI (kg/m^2^)					TPI (kg/m^3^)					BSA (cm^2^)				
	r	r^2^	SEE	cte	b	r	r^2^	SEE	cte	b	r	r^2^	SEE	cte	b
	Males														
HGSR (kg/f)	0.35	0.13	5.34	2.54	0.53	0.01	0.00	5.78	12.53	0.02	0.66	0.43	4.37	−5.29	15.08
HGSL (kg/f)	0.35	0.12	5.13	2.63	0.5	0.01	0.00	5.59	12.16	0.02	0.64	0.41	4.28	−4.77	14.34
HJ (cm)	0.05	0,00	26.48	117.55	−0.32	0.29	0.08	25.45	155.07	−3.09	0.26	0.07	25.68	78.28	27.63
Agility 5 × 10(seg)	0.00	0.00	3.43	24.65	0,00	0.26	0.07	3.32	19.63	0.35	0.31	0.1	3.26	29.76	−4.30
6MWT (m)	0.01	0.00	75.88	568.68	0.17	0.24	0.06	73.54	675.69	−7.37	0.3	0.09	72.24	461.5	91.93
	Females														
HGSR (kg/f)	0.27	0.07	4.84	5.24	0.32	0.00	0.00	5.02	11.13	−0.01	0.64	0.41	3.86	−5.29	15.08
HGSL (kg/f)	0.27	0.07	4.75	4.71	0.31	0,00	0.00	4.93	10.35	0.00	0.63	0.4	3.82	−5.05	13.89
HJ (cm)	0.06	0.00	20.96	93.11	0.28	0.14	0.02	20.81	110	−0.86	0.36	0.13	19.62	61.11	33.34
Agility 5 × 10(seg)	−0.05	0.00	3.23	27.08	−0.04	0.15	0.02	3.19	24.32	0.15	0.36	0.13	3.01	32.33	−5.34
6MWT (m)	0.05	0.00	66.86	578.65	−0.81	0.20	0.04	65.59	619.19	−4.01	0.21	0.05	65.41	493	63.31

HGSR, Right hand grip strength; HGSL, Left hand grip strength; HJ, Horizontal jump; 6MWT, 6-minute walk test; SEE, Standard error of estimation; BMI, Body mass index; TPI, Triponderal index; BSA, Body surface area.

Table 3, Table 4 show the percentile distribution of body size (BSA) and four physical tests by age and in both sexes. A progressive improvement in BSA is observed as chronological age increases. It can also be seen that as age increases, the values of the physical tests improve the performance in HGS and HJ, decrease the time in the round-trip running test (5 m x 10 repetitions), and increase the number of meters in the 6MWT.

**Table 3:** Percentile distribution of body size (BSA), HGS, HJ, agility and 6MWT of children 6 to 12 years of age from high altitude in Peru.

Age	L	M	S	P3	P5	P10	P15	P25	P50	P75	P85	P90	P95	P97
	BSA (cm^2^)													
6	−0.02	0.88	0.00	0.73	0.74	0.77	0.78	0.81	0.88	0.96	1.01	1.06	1.13	1.19
7	−0.02	0.95	0.00	0.77	0.79	0.82	0.84	0.87	0.95	1.04	1.10	1.14	1.22	1.28
8	−0.01	1.04	0.00	0.83	0.85	0.89	0.91	0.95	1.04	1.14	1.21	1.26	1.35	1.41
9	−0.01	1.12	0.00	0.88	0.90	0.94	0.97	1.02	1.12	1.24	1.31	1.37	1.46	1.52
10	−0.01	1.20	0.00	0.93	0.96	1.00	1.04	1.09	1.20	1.32	1.40	1.46	1.55	1.61
11	0.00	1.29	0.00	1.00	1.03	1.08	1.11	1.17	1.29	1.42	1.50	1.56	1.65	1.71
12	0.00	1.38	0.00	1.08	1.11	1.16	1.20	1.26	1.38	1.52	1.60	1.66	1.75	1.82
	HGSR (kg/f)													
6	0.01	6.30	0.00	3.2	3.5	4.1	4.5	5.1	6.3	7.5	8.2	8.7	9.5	9.9
7	0.01	7.10	0.00	3.8	4.2	4.7	5.2	5.8	7.1	8.6	9.5	10.0	11.0	11.6
8	0.00	8.50	0.00	4.8	5.1	5.8	6.2	7.0	8.5	10.3	11.4	12.1	13.3	14.2
9	0.00	10.30	0.00	6.1	6.5	7.2	7.7	8.6	10.3	12.5	13.8	14.8	16.4	17.6
10	0.00	12.50	0.00	7.6	8.1	8.9	9.4	10.4	12.5	15.0	16.6	17.8	19.8	21.2
11	0.00	14.90	0.00	9.1	9.7	10.6	11.3	12.4	14.9	17.9	19.8	21.2	23.5	25.2
12	0.00	18.00	0.00	11.0	11.7	12.8	13.7	15.0	18.0	21.6	24.0	25.7	28.5	30.6
	HGSL (kg/f)													
6	0.01	6.20	0.00	3.4	3.7	4.2	4.5	5.1	6.2	7.4	8.0	8.5	9.3	9.8
7	0.00	6.90	0.00	3.8	4.1	4.7	5.1	5.7	6.9	8.4	9.2	9.8	10.7	11.3
8	0.00	8.20	0.00	4.6	4.9	5.6	6.0	6.7	8.2	10.0	11.1	11.8	13.0	13.9
9	0.00	10.00	0.00	5.8	6.2	6.9	7.4	8.2	10.0	12.2	13.6	14.6	16.2	17.3
10	0.00	12.30	0.00	7.2	7.7	8.5	9.1	10.1	12.3	14.9	16.5	17.7	19.7	21.0
11	0.00	14.40	0.00	8.5	9.1	10.1	10.8	12.0	14.4	17.4	19.2	20.6	22.7	24.2
12	0.00	17.60	0.00	10.1	10.8	12.1	13.0	14.5	17.6	21.1	23.2	24.7	27.1	28.8
	HJ (cm)													
6	0.70	86.40	0.18	59.0	62.3	67.4	70.9	76.2	86.4	96.9	102.7	106.7	112.7	116.6
7	0.64	92.90	0.18	63.9	67.3	72.7	76.4	82.0	92.9	104.3	110.5	114.9	121.4	125.7
8	0.64	98.70	0.18	68.4	71.9	77.5	81.4	87.3	98.7	110.7	117.5	122.1	129.1	133.8
9	0.64	105.80	0.18	73.9	77.6	83.4	87.5	93.7	105.8	118.7	125.9	131.0	138.6	143.7
10	0.64	114.20	0.17	80.2	84.1	90.4	94.7	101.3	114.2	127.9	135.6	141.0	149.1	154.4
11	0.64	120.80	0.17	85.0	89.2	95.8	100.4	107.3	120.8	135.1	143.0	148.5	156.8	162.3
12	0.64	131.20	0.17	92.4	96.9	104.1	109.0	116.5	131.2	146.6	155.2	161.2	170.3	176.3
	Agility 5 × 10 (seg)													
6	−0.75	29.20	0.12	37.5	36.2	34.4	33.3	31.8	29.2	27.0	25.9	25.2	24.2	23.6
7	−1.18	27.40	0.11	35.0	33.8	32.2	31.1	29.7	27.4	25.5	24.6	24.0	23.2	22.7
8	−1.60	25.80	0.10	32.7	31.6	30.0	29.1	27.8	25.8	24.2	23.4	22.9	22.2	21.8
9	−1.79	24.50	0.10	30.5	29.5	28.1	27.3	26.2	24.5	23.0	22.3	21.9	21.3	20.9
10	−1.86	23.50	0.09	28.8	28.0	26.8	26.0	25.1	23.5	22.1	21.5	21.1	20.6	20.2
11	−1.91	22.90	0.09	27.8	27.0	25.9	25.2	24.3	22.9	21.6	21.1	20.7	20.2	19.9
12	−1.81	21.90	0.08	26.2	25.5	24.6	24.0	23.2	21.9	20.8	20.2	19.9	19.4	19.1
	6MWT (m)													
6	1.87	493.40	0.10	391.5	405.5	426.2	439.8	459.1	493.4	525.7	542.3	553.3	569.3	579.5
7	2.16	515.30	0.10	402.9	419.0	442.4	457.4	478.5	515.3	549.3	566.6	578.0	594.3	604.7
8	2.39	540.60	0.10	417.5	435.6	461.7	478.2	501.2	540.6	576.3	594.2	606.0	622.8	633.4
9	2.38	560.30	0.10	432.1	451.0	478.2	495.3	519.3	560.3	597.6	616.3	628.6	646.1	657.2
10	1.92	584.10	0.10	456.1	473.9	500.2	517.2	541.4	584.1	624.0	644.5	658.1	677.7	690.2
11	1.32	606.40	0.11	479.1	495.6	520.7	537.4	561.8	606.4	649.9	672.9	688.3	711.0	725.6
12	0.63	623.00	0.11	498.6	513.7	537.1	553.2	577.2	623.0	670.0	695.8	713.4	739.9	757.3

P, Percentile, L (Lambda, Box-Cox transformation), M (Mu) median of the distribution and S (Sigma) coefficient of variation.

**Table 4:** Percentile distribution of body size (BSA). HGS. HJ. agility and 6MWT of girls aged 6 to 12 years from high altitude in Peru.

Age	L	M	S	P3	P5	P10	P15	P25	P50	P75	P85	P90	P95	P97
	BSA (cm^2^)													
6	−0.03	0.82	0.00	0.69	0.70	0.72	0.74	0.76	0.82	0.89	0.95	0.99	1.08	1.15
7	−0.02	0.90	0.00	0.75	0.77	0.79	0.81	0.84	0.90	0.99	1.05	1.09	1.17	1.24
8	−0.02	0.99	0.00	0.81	0.83	0.86	0.88	0.91	0.99	1.08	1.14	1.19	1.27	1.33
9	−0.01	1.08	0.00	0.88	0.90	0.93	0.96	1.00	1.08	1.18	1.25	1.30	1.38	1.44
10	−0.01	1.19	0.00	0.95	0.98	1.02	1.05	1.10	1.19	1.31	1.37	1.42	1.50	1.55
11	0.00	1.29	0.00	1.01	1.04	1.09	1.13	1.19	1.29	1.41	1.48	1.52	1.60	1.64
12	0.01	1.39	0.00	1.05	1.09	1.16	1.20	1.27	1.39	1.51	1.57	1.62	1.68	1.72
	HGSR (kg/f)													
6	0.005	5.800	0.003	2.8	3.1	3.6	4.0	4.6	5.8	7.2	7.9	8.5	9.3	9.9
7	0.003	7.100	0.003	3.6	4.0	4.6	5.0	5.7	7.1	8.7	9.6	10.3	11.4	12.1
8	0.003	8.300	0.003	4.5	4.8	5.5	6.0	6.7	8.3	10.2	11.3	12.1	13.3	14.2
9	0.004	10.300	0.003	5.5	6.0	6.8	7.4	8.4	10.3	12.5	13.7	14.6	16.1	17.0
10	0.006	12.900	0.003	7.0	7.6	8.7	9.5	10.6	12.9	15.5	16.9	17.9	19.5	20.5
11	0.008	15.500	0.003	8.6	9.5	10.7	11.6	13.0	15.5	18.3	19.7	20.8	22.3	23.3
12	0.009	17.800	0.002	10.5	11.4	12.8	13.8	15.2	17.8	20.5	21.9	22.9	24.4	25.3
	HGSL (kg/f)													
6	0.00	5.40	0.003	3.1	3.4	3.7	4.0	4.5	5.4	6.4	7.0	7.4	8.1	8.6
7	0.00	6.50	0.003	3.7	4.0	4.5	4.8	5.4	6.5	7.8	8.6	9.2	10.1	10.7
8	−0.01	7.60	0.003	4.9	5.1	5.5	5.8	6.4	7.6	9.4	10.7	11.8	13.8	15.5
9	0.00	9.40	0.003	5.1	5.6	6.3	6.9	7.7	9.4	11.4	12.6	13.4	14.7	15.6
10	0.01	12.40	0.003	6.2	6.9	8.0	8.8	10.0	12.4	14.9	16.2	17.2	18.6	19.6
11	0.01	15.00	0.003	7.5	8.4	9.8	10.8	12.3	15.0	17.8	19.2	20.3	21.8	22.7
12	0.01	16.70	0.002	8.1	9.4	11.1	12.3	13.9	16.7	19.4	20.7	21.7	23.0	23.8
	HJ (cm)													
6	1.63	77.10	0.202	42.7	47.8	55.1	59.7	66.1	77.1	87.2	92.3	95.7	100.5	103.6
7	1.22	84.30	0.195	51.8	56.1	62.5	66.8	73.0	84.3	95.3	101.0	104.9	110.6	114.2
8	0.84	92.20	0.186	60.9	64.7	70.6	74.7	80.7	92.2	103.9	110.3	114.6	121.1	125.4
9	0.58	97.40	0.174	67.8	71.2	76.7	80.5	86.2	97.4	109.1	115.6	120.1	126.9	131.4
10	0.55	105.30	0.161	75.6	79.1	84.6	88.4	94.1	105.3	116.9	123.4	127.9	134.7	139.2
11	0.85	114.30	0.148	83.2	87.0	93.0	97.0	103.0	114.3	125.8	132.0	136.2	142.5	146.7
12	1.32	116.90	0.135	85.8	89.9	96.1	100.2	106.1	116.9	127.4	132.9	136.6	142.0	145.5
	Agility 5 × 10 (seg)													
6	−2.32	29.30	0.109	38.7	37.0	34.7	33.4	31.8	29.3	27.4	26.5	26.0	25.3	24.8
7	−1.91	28.50	0.100	36.0	34.7	33.0	32.0	30.6	28.5	26.7	25.9	25.4	24.7	24.3
8	−1.35	26.90	0.092	32.8	31.9	30.6	29.8	28.7	26.9	25.4	24.6	24.2	23.5	23.1
9	−1.05	25.70	0.085	30.6	29.8	28.8	28.1	27.2	25.7	24.3	23.6	23.2	22.5	22.1
10	−1.03	24.70	0.080	29.1	28.4	27.5	26.9	26.1	24.7	23.4	22.8	22.4	21.8	21.5
11	−1.47	24.10	0.076	28.3	27.7	26.8	26.2	25.4	24.1	22.9	22.4	22.0	21.5	21.2
12	−1.79	23.70	0.073	27.7	27.1	26.2	25.7	24.9	23.7	22.6	22.1	21.7	21.3	21.0
	6MWT (m)													
6	−0.32	499.80	0.101	415.9	425.4	440.5	451.1	467.4	499.8	535.3	555.8	570.2	592.5	607.6
7	0.87	533.20	0.098	435.9	447.9	466.6	479.3	498.0	533.2	568.8	588.0	601.0	620.4	633.1
8	2.06	556.30	0.097	442.2	458.2	481.7	496.9	518.4	556.3	591.6	609.7	621.6	638.8	649.8
9	2.99	573.90	0.097	440.2	461.5	490.9	508.9	533.4	573.9	609.3	626.8	638.0	654.1	664.1
10	3.63	598.90	0.097	444.8	472.4	508.0	528.8	556.0	598.9	635.0	652.4	663.4	679.0	688.6
11	3.98	607.60	0.094	448.5	478.6	516.0	537.4	564.9	607.6	642.8	659.6	670.2	685.2	694.4
12	4.21	593.70	0.09	442.6	471.6	507.4	527.7	553.7	593.7	626.5	642.1	652.0	665.8	674.3

P, Percentile, L (Lambda, Box-Cox transformation), M (Mu) median of the distribution and S (Sigma) coefficient of variation.

## Discussion

The initial objective of the study was to verify the applicability of anthropometric indicators (BMI, TPI, and BSA) to predict physical fitness in schoolchildren living in a high-altitude region of Peru. The results of the study have shown that BSA is the best overall predictor of physical fitness relative to BMI and TPI. These findings highlight the importance of BSA as a key indicator of physical performance in schoolchildren. It outperformed BMI and TPI in its predictive ability in the HGS (right and left), HJ, agility, and 6MWT tests.

These findings suggest that the BSA should be considered as a relevant criterion in the assessment and classification of physical fitness in this population of schoolchildren aged 6 to 12 years living at high altitude.

The BSA provides a more accurate representation of body composition, as it considers both height and weight with allometric correction. Weight is approximately proportional to a subject's height. Therefore, the product of weight (a) × height (b) can be manipulated by an appropriate constant to obtain a stable value that approximates weight.

This allows a more comprehensive assessment of metabolic mass than body weight, as it is less affected by abnormal adipose mass. Unlike BMI and TPI, which can be affected by fat and muscle distribution, BSA is more closely associated with muscle mass and metabolism, which is crucial for physical performance.

In fact, some recent studies have reported that BSA is an excellent predictor of fat-free mass in young people and adults.^20^,^21^ Therefore, its use and application in children living at high altitude in Peru becomes fundamental in the evaluation of the morphological component. Therefore, incorporating the BSA together with physical tests can provide valuable information for designing training programs and monitoring progress in schoolchildren aged 6 to 12 years.

Overall, BSA can provide a more complete and useful insight into understanding how children living at high altitude can optimize their physical capacity according to body size. This information is crucial for pediatric populations living in high-altitude geographic regions, where BMI is often used as an indicator of nutritional status. Although previous studies have suggested great limitations due to the low stature in relation to international references.^9^,^10^

The second objective of the study was to propose reference values to assess physical fitness in children living at high altitude in Peru. For this purpose, the LMS method was used to represent the distribution of percentiles of body size (BSA), HGS (right and left), HJ, agility, and 6MWT.

In general, normative data from field fitness tests offer the opportunity to analyze and study health promotion and sports skills development at the school level.^22^ In the specific case of Peru, the Ministry of Education does not specify the use and/or application of a battery to assess the physical fitness of schoolchildren. But it does, in its guidelines, state a clear and structured progression of achievement objectives covering all levels of schooling, highlighting the balanced development of the body and health.^23^

From that perspective, the percentiles proposed here for body size and four physical tests were designed for primary school children in a high-altitude region of Peru. These reference values were developed by age and sex and can be used for diagnostic purposes and evaluation of the physical fitness level of children aged 6 to 12 years.

To categorize performance levels, it is necessary to rely on some cut-off points. For this purpose, in this research, the authors use suggestions described by some recent studies.^24^,^25^ These investigations have reported, for example, that children below the 15th percentile (< p15) are characterized by warning signs of low level of performance. While those between p15 to p85 are considered adequate, and above > p85 are considered a high fitness level.

The use of fitness reference values in the school system can generate individual and/or collective advantages.^26^ Since schoolchildren below p15 are identified, physical education professionals can implement strategies and intervention programs to improve physical fitness. On the other hand, higher levels of physical fitness allow participation in a variety of physical activities and consequently decrease the risk of health problems.^27^,^28^

Therefore, to successfully monitor fitness levels over time, it is essential to have up-to-date normative data ^29^. In this sense, the reference values proposed in this study are a valuable tool for the school population living at high altitudes. This tool can be useful for health professionals, as well as for parents and physical education teachers. In addition, some studies have characterized fitness percentiles as a simple, safe, and low-cost tool to examine various health indicators.^30^

In sum, monitoring and measuring the progression of physical fitness in schoolchildren in various geographic regions is extremely relevant. Especially in these times where fitness levels have been gradually deteriorating.^2^ Therefore, it is essential to systematically and frequently monitor these indicators using BSA as a physiological estimator of body size (related to energy expenditure and exchange surface area). This would allow discrepancies between size and functional capacity that BMI and TPI do not detect to be identified. Likewise, when applied to clinical practice and the school environment, the use of BSA could facilitate allometric adjustment in physical tests, allowing for a more accurate interpretation of children's physical performance by taking into account actual body size and not just weight and height, respectively.

The present study has certain limitations, as, for example, in relation to the age range, it was not possible to evaluate the physical fitness of secondary school children (13 to 17 years). Therefore, future studies should fill this gap. On the other hand, the selection of the sample used in the study was non-probabilistic, and the design used was cross-sectional. This limits the generalization of the results, and due to the type of study, it is not possible to establish causal relationships. In addition, a standard method for analyzing body composition is necessary, since both BSA, BMI, and TPI are estimated using formulas. This may generate some variations in the results of this study. Therefore, future studies should consider these aspects to strengthen their methodological designs.

The study presents some strengths that should be recognized, since it is one of the first studies that proposes to consider BSA as part of the morphological component. In addition, the proposed percentiles evidence updated data, which allows monitoring fitness levels in high altitude primary school children. These results can serve as a baseline for future comparisons and possible secular trend analysis.

## Conclusion

BSA was shown to be the best predictor of physical fitness in schoolchildren living in high-altitude areas in Peru, far surpassing BMI and TPI. This finding highlights the physiological value of BSA as an indicator of body size and efficiency, providing novel information in a little-explored context. Reference values applicable in clinical and school settings were also proposed, allowing for more accurate assessment of physical fitness according to age and sex.

## Authors’ contributions

Conceived and designed the experiments: Marco Cossio-Bolaños, Rossana Gómez Campos.

Performed the experiments: Jose Fuentes-Lopez, Dony Mamani Velasquez, Eliseny Vargas Ramos, Estanislao Pacompia Cari.

Analyzed the data: Marco Cossio-Bolaños, Rossana Gómez Campos, Rubén Vidal Espinoza, Wilbert Cossio Bolaños.

Contributed reagents/materials/analysis tools: Jose Fuentes-Lopez, Eliseny Vargas Ramos.

Wrote the paper: Marco Cossio-Bolaños, Rossana Gómez Campos, Rubén Vidal Espinoza, Danilo R. Silva.

## Funding

This research did not receive any specific grant from funding agencies in the public, commercial, or not-for-profit sectors.

## Data availability

The data supporting the findings of this study are openly available in the Figshare repository at http://dx.doi.org/10.6084/m9.figshare.30862223.

## Conflicts of interest

The authors declare no conflicts of interest.

## References

[1] Iglesias-Soler E., Rúa-Alonso M., Rial-Vázquez J., Lete-Lasa J.R., Clavel I., Giráldez-García M.A. (2021). Percentiles and principal component analysis of physical fitness from a big sample of children and adolescents aged 6-18 years: the DAFIS Project. Front Psychol.

[2] Mamen A., Fredriksen P.M. (2018). Anthropometric measures as fitness indicators in primary school children: the Health Oriented Pedagogical Project (HOPP). Scand J Public Health.

[3] Bouchard C.E., Shephard R.J., Stephens T.E. (1994). International consensus symposium on physical activity, fitness, and health, 2nd, May 1992.

[4] Pate R., Oria M., Pillsbury L. (2012). Committee on fitness measures and health outcomes in youth; food and nutrition board; institute of medicine. fitness measures and health outcomes in youth.

[5] Kolimechkov S. (2017). Evaluación de la aptitud física en niños y adolescentes. Una revisión sistemática. EJPESS.

[6] Gupta P.M., Wieck E., Conkle J., Betters K.A., Cooley A., Yamasaki S. (2020). Improving assessment of child growth in a pediatric hospital setting. BMC Pediatr.

[7] Piqueras P., Ballester A., Durá-Gil J.V., Martinez-Hervas S., Redón J., Real J.T. (2021). Anthropometric indicators as a tool for diagnosis of obesity and other health risk factors: a literature review. Front Psychol.

[8] Lopes V.P., Malina R.M., Gomez-Campos R., Cossio-Bolaños M., Arruda M., Hobold E. (2019). Body mass index and physical fitness in Brazilian adolescents. J Pediatr (Rio J).

[9] Cossio-Bolaños M., Vidal-Espinoza R., Albornoz C.U., Fuentes-Lopez J., Sánchez-Macedo L., Andruske C.L. (2022). Relationship between the body mass index and the ponderal index with physical fitness in adolescent students. BMC Pediatr.

[10] Cossio-Bolaños M.A., Maria T.S., Campos R.G., Pascoal E.H., Hespanhol J.E., Arruda M.D. (2012). The use of World Health Organization growth curves in children and adolescents that live in regions of moderate altitude. Rev Paul Pediatr.

[11] Cossio Bolaños M.A., Viveros Flores A., Eduardo Hespanhol J., Camargo C., Gomez Campos R. (2014). Aplicabilidad del IMC en adolescentes escolares que viven a moderada altitud del Perú [Applicability of BMI in adolescent students living at moderate altitude of Perú]. Nutr Hosp.

[12] Ma X., Mao Y., Wang J., Zewangzhandui Wang X (2022). Anthropometric indices, body function, and physical fitness reference values for Tibetan ethnic children aged 6-17 residing at 3650 meters above sea level. Front Nutr.

[13] Feber J., Krásničanová H., Preedy V. (2012). Handbook of anthropometry: physical measures of human form in health and disease.

[14] Ross W.D., Marfell-Jones M., Clarys-Robion J.P, MacDougall J.D., Wenger H.A., Green H.J. (1983). Physiological testing of the high-performance athlete.

[15] Haycock G.B., Schwartz G.J., Wisotsky D.H. (1978). Geometric method for measuring body surface area: a height-weight formula validated in infants, children, and adults. J Pediatr.

[16] Richards L.G., Olson B., Palmiter-Thomas P. (1996). How forearm position affects grip strength. Am J Occup Ther.

[17] Castro-Piñero J., Ortega F.B., Artero E.G., Girela-Rejón M.J., Mora J., Sjöström M. (2010). Assessing muscular strength in youth: usefulness of standing long jump as a general index of muscular fitness. J Strength Cond Res.

[18] Verschuren O., Takken T., Ketelaar M., Gorter J.W., Helders P.J. (2007). Reliability for running tests for measuring agility and anaerobic muscle power in children and adolescents with cerebral palsy. Pediatr Phys Ther.

[19] (2002). ATS Committee on Proficiency Standards for Clinical Pulmonary Function Laboratories. ATS statement: guidelines for the six-minute walk test. Am J Respir Crit Care Med.

[20] Zanforlini B.M., Alessi A., Pontarin A., De Rui M., Zoccarato F., Seccia D.M. (2021). Association of body surface area with fat mass, free fat mass and total weight in healthy individuals, and implications for the dosage of cytotoxic drugs. Clin Nutr ESPEN.

[21] García-Hilares D., Vidal Espinoza R., De la Torre Choque C., Equivel Segura H., Baquerizo Sedano L., Vidal-Fernandez N. (2024). Indicadores antropométricos como predictores de la masa libre de grasa en basquetbolistas universitarios 3 x 3. Nutr Clín Diet Hosp.

[22] Golle K., Muehlbauer T., Wick D., Granacher U. (2015). Physical fitness percentiles of German children aged 9-12 years: findings from a longitudinal study. PLoS One.

[23] Ministerio de Educación Perú (MINEDU) (2010). Orientaciones para el Trabajo Pedagógico del Área de Educación Física. Cuarta edición.

[24] Hobold E., Pires-Lopes V., Gómez-Campos R., de Arruda M., Andruske C.L., Pacheco-Carrillo J. (2017). Reference standards to assess physical fitness of children and adolescents of Brazil: an approach to the students of the Lake Itaipú region-Brazil. Peer J.

[25] Gómez-Campos R., Andruske C.L., Arruda M., Sulla-Torres J., Pacheco-Carrillo J., Urra-Albornoz C. (2018). Normative data for handgrip strength in children and adolescents in the Maule Region, Chile: evaluation based on chronological and biological age. PLoS One.

[26] Galvani C., Togni F., Puci M.V., Vandoni M., Correale L., Codella R. (2024). Health-related field-based fitness tests: normative values for Italian primary school children. J Funct Morphol Kinesiol.

[27] Boraczynski M., Boraczynski T., Podstawski R., Mankowski S., Choszcz D., Honkanen A. (2015). Physical fitness classification standards for Polish early education teachers. S Afr J Res Sport Phys Educ Recreat.

[28] Marttinen R., Fredrick R.N., Silverman S.S (2018). Middle school students’ free-living physical activity on physical education days, non-physical education days, and weekends. Monten J Sports Sci Med.

[29] Niessner C., Utesch T., Oriwol D., Hanssen-Doose A., Schmidt S.C., Woll A. (2020).

[30] Przednowek K.H., Niewczas M., Wójcik Ł., Paśko W., Iskra J., Przednowek K. (2021). Physical fitness percentiles of Polish children aged 4-7 years. Sci Rep.

